# Understanding the research landscape of over-the-counter herbal products, dietary supplements, and medications evaluated for depressive symptoms in adults: a scoping review

**DOI:** 10.3389/fphar.2025.1609605

**Published:** 2025-07-15

**Authors:** Rachael Frost, Aiman Zamri, Silvy Mathew, Adriana Salame, Cini Bhanu, Sukvinder K. Bhamra, Juan Carlos Bazo-Alvarez, Michael Heinrich, Kate Walters

**Affiliations:** ^1^ School of Public and Allied Health, Liverpool John Moores University, Liverpool, United Kingdom; ^2^ Department of Primary Care and Population Health, University College London, London, United Kingdom; ^3^ Division of Medicine, University College London, London, United Kingdom; ^4^ Medway School of Pharmacy, University of Kent, Kent, United Kingdom; ^5^ Escuela de Medicina, Universidad Cesar Vallejo, Trujillo, Peru; ^6^ UCL School of Pharmacy, University College London, London, United Kingdom; ^7^ China Medical University, Taichung, Taiwan

**Keywords:** depression, scoping review, herbal medicine, dietary (food) supplements, major depressive disorder

## Abstract

**Background:**

Over-the-counter (OTC) products such as herbal medical products (HMPs) or dietary supplements are a valued part of preventative and supportive self-care for depressive symptoms, but there is a wide array of products available, with differing levels of clinical evidence. It is unclear what the optimal directions for future research in this field are.

**Aim:**

We aimed to explore the size and nature of the evidence base available for OTC products for depression in adults aged 18–60.

**Methods:**

We carried out a scoping review following Joanna Briggs Institute guidance. We searched MEDLINE, Embase, PsycINFO, AMED, and CENTRAL from inception to December 2022, and 10% of the results were screened by two authors and the remainder by one author. We included randomised controlled trials of products commonly available OTC in multiple countries in participants with symptoms or a diagnosis of depression. Results were narratively summarised by the product and volume of evidence available.

**Results:**

Out of 23,933 records found, we screened 1,367 full texts and included 209 trials. The largest volume of evidence was for omega-3s, St John’s Wort, saffron, probiotics, and vitamin D. Among a range of herbal medical products with promising evidence, those most commonly used and thus warranting further research were lavender, lemon balm, chamomile, and Echium. For 41 products, we found only single trials. Few products presented safety issues, whether used alone or adjunctively with antidepressants.

**Conclusion:**

Products with limited but promising evidence included folic acid, lavender, zinc, tryptophan, Rhodiola, and lemon balm, and future research should focus on these products. There is a need for further evaluation of herbal medical products as adjuncts to antidepressants and for exploring their potential benefits when used adjunctively with psychological therapies to support a more integrative approach. Safety reporting in these trials needs to be further improved.

**Systematic Review Registration:**

https://osf.io/rkm57/.

## Introduction

Depression is common—the prevalence of major depressive disorder (MDD) varies between 2% and 21%, depending on the country and measure used (Gutiérrez-Rojas et al., 2020), and prevalence is increasing over time (Moreno-Agostino et al., 2021). MDD is defined according to the DSM-V as the presence of five or more out of nine symptoms experienced frequently within the same two-week period, including depressed mood and loss of pleasure, in addition to other symptoms such as changes in appetite, insomnia, and fatigue. Symptoms must also cause distress or impairment, with the severity of depression determined by the level of impairment, and the condition cannot be better explained by another cause (Uher et al., 2014).

In the United Kingdom (UK), 11.3% report mild depressive symptoms, 4.2% report moderate depressive symptoms, and 3.3% report severe depressive symptoms (Arias De La Torre et al., 2021). MDD is associated with multiple interacting pathogenic factors, including genetics, stressful life events, and chronic diseases (Cui et al., 2024). Underlying pathophysiological theories for depression include 1) deficiencies in monoamine neurotransmitters (e.g., serotonin and dopamine), 2) an increase in hypothalamic–pituitary–adrenal (HPA) axis activity, increasing production of glucocorticoids, which provide negative feedback to the limbic system, hypothalamus, and pituitary, and 3) low-grade inflammation that is self-sustaining, with the presence of reactive oxygen species further triggering inflammatory cytokines, which compromise neuroendocrine activities (Cui et al., 2024). There may also be impaired neurogenesis due to reduced brain-derived neurotrophic factor (BDNF) (Dobrek and Głowacka, 2023) or the effects from the gut microbiota on the function of the HPA axis (Dobrek and Głowacka, 2023).

Depression has a strong impact on individuals, reducing the quality of life and increasing the risk of physical health conditions such as cardiovascular disease and diabetes (Graham et al., 2020; Harshfield et al., 2020). There is also a widespread societal impact as depression increases the risk of sickness absence from employment (Amiri and Behnezhad, 2021). Globally, 16% of disability-adjusted life years can be attributed to depression, reflecting an economic cost of USD$ 4.7 trillion (Arias et al., 2022). There is, therefore, a strong need to find effective methods to prevent, manage, and reduce depressive symptoms in adults.

In the UK, the National Institute for Health and Care Excellence (NICE) guidelines recommend discussions of psychological treatments (e.g., group and individual psychological therapies and guided self-help), exercise, and pharmacological treatment options (e.g., antidepressants), with a decision made jointly with the patient as to which is the most appropriate, considering the least intrusive and least resource-intensive options first (National Institute for Health and Care Excellence, 2022). However, it is estimated that in Great Britain, only 13.4% of individuals reporting depressive symptoms receive some form of treatment (Smits and Huijts, 2015), with particular challenges in accessing psychological therapies due to long waiting times (Baker and Kirk-Wade, 2024). Although antidepressants are more easily accessible, they require help-seeking with a medical practitioner in order to receive them, and views and experiences of their perceived effectiveness, desirability, and side effects are mixed (Crowe et al., 2023).

Consequently, it is unsurprising that prior to, or whilst receiving treatment, people often find ways to self-manage depressive symptoms, most commonly using herbal medical products (HMPs) and vitamins and minerals rather than practitioner-directed modalities (Solomon and Adams, 2015). Homoeopathic preparations are also a possible approach, with one of the most common reasons for using over-the-counter (OTC) homoeopathic preparations in the UK being psychological problems (Reid, 2002). Although there are no specific OTC medicines licensed for depressive symptoms, it is possible that OTC medicines are also used more frequently to mitigate depressive symptoms or symptoms linked to depression (e.g., insomnia).

It is suggested that natural products, such as HMPs or nutrients, may help manage depression through various mechanisms, including inhibiting inflammation, ameliorating oxidative stress, changing the microbiota–gut–brain axis, suppressing hyperactivity in the hypothalamic–pituitary–adrenal axis, and regulating neurotransmitters (Wu et al., 2022). These products are generally derived from different medical traditions and are now an important part of self-care in many countries, either regulated as medicines or supplements/botanicals. Qualitative studies suggest that products such as St John’s Wort can be viewed as more desirable due to perceptions of safety, naturalness, and greater ability to control usage (Pirotta et al., 2014), and they may be used as alternatives to, or in addition to, prescribed antidepressants. There is little mention in the NICE guidelines regarding OTC products for depression, apart from a recommendation not to advise St John’s Wort due to uncertainty about doses and the potential for interactions (National Institute for Health and Care Excellence, 2022).

There has been an abundance of evidence evaluating OTC products for depression over the last few decades, particularly products such as St John’s Wort and omega-3 supplements. These often have relatively conclusive separate systematic reviews and meta-analyses [e.g., St John’s Wort (Ng et al., 2017), saffron (Tóth et al., 2019), and omega-3s (Liao et al., 2019)]. Although this can provide a rigorous assessment of the strength and level of evidence available for each product and a single estimate of effect, it tends to focus the evidence base on a few key products and neglects the wider scope of other promising products that may offer future avenues for prevention and self-care of depressive symptoms.

Where reviews have previously attempted to summarise the wider evidence base beyond single products, they have often relied on narrative summaries (Sarris, 2018) or included only a limited number of studies (Kamat et al., 2023). It is, therefore, valuable to scope the evidence base as a whole, including ongoing trials, to understand where the literature is concentrated and where gaps exist. This allows future research directions to be clearly outlined, broadening the potential field for clinical studies in products for depressive symptoms beyond those with an already high level of evaluation. We, therefore, aimed to

1. Summarise the size and nature of the evidence base that assesses the effectiveness of OTC products for depression in adults aged 18–60.
2. Determine the areas with substantial evidence and those with gaps to identify directions for future research.

## Methods

Following Joanna Briggs Institute (JBI) guidance (Peters et al., 2020) and PRISMA guidance for scoping reviews (Tricco et al., 2018), we carried out a scoping review. The review formed part of a larger project summarising the available trial evidence for OTC products for depression, anxiety, and insomnia. It was prospectively registered on the Open Science Framework (https://osf.io/rkm57/). Due to the large volume of results, we synthesised the findings separately for each condition and grouped them according to whether the samples were adults (aged 18–60) or older adults to explore whether an age bias existed in these trials. Two public contributors were involved in developing the review questions, designing the review, and selecting which products to include.

We searched five databases (MEDLINE, Embase, PsycINFO, AMED, and CENTRAL) from inception to December 2022. The CENTRAL searches included trial registry entries. Search terms were grouped into OTC product terms, mental health terms, and randomised controlled trial (RCT) filters (where applicable) and combined using Boolean operators (see Supplementary Material 1). Given the multitude of possible products, product categories rather than individual names were used in searches. The research team piloted searches in MEDLINE and Embase and refined these as needed.

Search results were imported into Rayyan (Ouzzani et al., 2016) after deduplication. Due to the large volume of results, dual title and abstract screening was undertaken for 10% of titles and abstracts by pairs of reviewers (RF, SM, SU, VT, and AS). Reviewers were provided with explicit instructions to maximise consistency, and each pair conducted three batches of screening; discussions and decisions were agreed upon by all five reviewers, and inclusion criteria refined as needed. Only when agreement was 85%–90% between all pairs of reviewers (after the third batch) were the remaining studies screened by a single reviewer. Studies deemed unclear were moved to the full-text stage. The same process was carried out for full-text screening, with decisions documented in MS Excel, and unclear studies at this stage were screened by a second reviewer. As part of screening, studies were labelled according to whether they focussed on anxiety, depression, insomnia, or a combination and whether their sample included people aged 18–60 or 60+. In this article, studies relating to depression in adults aged 18–60 are synthesised.

We included randomised controlled trials (including parallel, factorial, and crossover trials) evaluating products that met the following criteria:

• were likely to be available OTC in a number of countries globally.
• consisted of single chemical medicines, HMPs, homoeopathic products, and/or dietary supplements taken orally. The list of potential products was developed in consultation with our patient and public involvement representatives and so included a wide range of products that may be used by consumers, including homoeopathic products.
• were evaluated alone or as an adjunct treatment.
• included a comparator, without restriction on whether this was a non-pharmacological intervention, prescription medication, another OTC product, no treatment, or placebo.
• were used for at least 1 week.
• typically did not require practitioner input or individualisation (e.g., some traditional Chinese medicine and homoeopathic interventions).

We included studies where the sample mean ages fell between 18 and 60 years and where participants had depressive symptoms established at baseline—either by meeting a threshold on an established depression questionnaire or with a diagnosis of minor or major depressive disorder. Studies that included people with depression and a comorbid condition were eligible, but those focussing on other mental health conditions (e.g., bipolar disorder, dementia, and substance abuse) were excluded. Studies needed to include depressive symptoms or depression remission as an outcome measure. We included trials from any time period and in any language, using Google Lens or a multilingual colleague where available for non-English texts. Subgroup analyses and grey literature presenting result data (e.g., theses) were excluded.

For all identified trial registry entries, published protocols, and conference abstracts, during the period of April–June 2024 we endeavoured to locate the main trial publication through trial number searches, title searches, and “cited by” functions (published protocols only) and by reviewing named author publication lists. Where full texts could not be located, we summarised ongoing trials and published protocols (see Supplementary Material 2) but not conference abstracts. Full texts located through these methods were screened by one author (RF) and were included and extracted if eligible. Reference list screening of relevant reviews was undertaken by SM for 10% (n = 200) reviews; as few new studies were detected and resources were limited, no further reference lists were screened after this point.

Data were extracted using a data extraction form designed according to the JBI data extraction template, including study details such as country, setting, sample size, inclusion criteria, participant characteristics, product characteristics, comparators, effectiveness outcomes for depression, and safety outcomes. Data were extracted by a reviewer (AZ), who also conducted a second eligibility check of included studies. Codings/groupings were applied by RF, who checked any uncertainties by consulting the original papers if needed and confirmed the exclusion of any ineligible studies. RF also extracted data from studies identified through trial registry and conference abstract follow-up. For plants, both common and Latin names were extracted where reported; however, where these were not reported, we avoided imposing potentially incorrect names and instead used the name(s) listed in the study.

To synthesise the data, we grouped products by overall and specific product type. We summarised the products evaluated, comparators used, comorbid conditions, and whether the products were used as adjuncts or evaluated alone. Due to the large volume of studies, we summarised effectiveness and safety data using vote counting. We classified effectiveness findings according to whether they showed significant effects at ≥ 1 timepoint on any depression trial outcome compared to each type of control treatment, and we classified safety findings by whether there were differences between groups in adverse events (see Table 1 for categorisations applied by RF). Only comparisons relevant to the review were included (e.g., prescribed antidepressant vs. placebo comparisons in a three-arm trial are not presented). We did not carry out a quality assessment as this was not the aim of scoping reviews, which provide a descriptive overview of the evidence base (Peters et al., 2020). We aimed to highlight areas with larger volumes of evidence and identify gaps within the evidence base needing further research.

**TABLE 1:** Classification of effectiveness and safety.

	Classification	Symbol	Definition
Effectiveness	Positive vs. placebo	+	Significantly better than placebo on ≥1 depression outcome
	Null vs. placebo	○	Null findings on all depression outcomes compared to placebo
	Positive vs. active	+	Significantly better than prescribed drug on ≥1 depression outcome
	Null vs. active	○	Not significantly different or non-inferior to a prescribed medication such as SSRIs on all depression outcomes
	Negative vs. active	—	Active drug shows significantly better outcome on ≥1 depression outcomes
	Positive vs. no treatment	+	Significantly better than no treatment ≥1 depression outcome—often compared as antidepressant with or without the OTC product as an adjunct
	Null vs. no treatment	○	Null findings on all depression outcomes compared to no treatment
	Positive vs. other OTC	+	Significantly better than another OTC preparation, e.g., another herbal medical product on ≥1 depression outcome
	Null vs. other OTC	○	Null findings on all depression outcomes compared to another OTC preparation, e.g., another herbal medical product
	Positive high vs. low dose	+	Significantly better than a lower dose of the product on ≥1 depression outcome at ≥1 timepoint
	Null high vs. low dose	○	Null findings on all depression outcomes when one dose is compared to a lower dose of the same product
	Unclear	?	Significance tests are not reported for between-group analysis
Safety	No safety concerns	**✔✔**	Similar rates between groups or higher rates of events in the control group
	Mild differences only	**✔**	Significant or substantial numeric differences in mild AEs between groups, e.g., higher frequency of heartburn
	Safety concerns	✗	Significant or substantial differences in serious adverse events between groups
	NR	NR	Not reported

## Results

**FIGURE 1:**
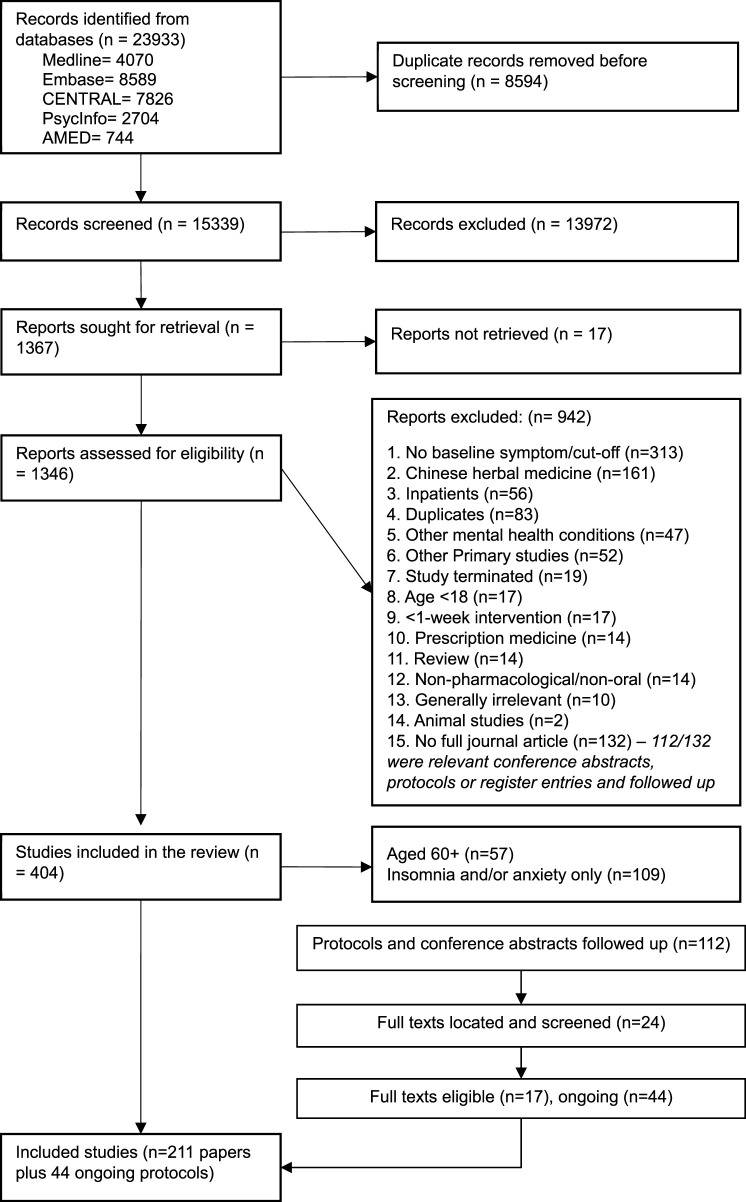
PRISMA flow diagram.

Figure 1 shows the flow of studies through this review. Out of 15,339 records screened, 13,972 were excluded on the basis of title and abstract, and 1,367 full texts were screened. One hundred and ninety papers relating to depression in adults aged 18–60 were included, with a further 16 identified from following up registered protocols and conference abstracts, providing a total of 209 included trials. Of these, 196 evaluated products for depression alone, 10 for depression and anxiety, 2 for depression and insomnia, and 1 for all three conditions. Tables 2–5 summarise all included studies with references.

**TABLE 2:** Products with substantive evidence.

Product	N studies (sample size range)	Dose and preparation	N as adjunct to other depression treatment	Comparator	N comorbid conditions	Effect on depression	Safety concern
Omega-3s (Rapaport et al., 2016; Rogers et al., 2008; Keshavarz et al., 2018; Nishi et al., 2020; Freeman et al., 2006; Ginty and Conklin, 2015; Meyer et al., 2013; Jahangard et al., 2018; Carney et al., 2009; Gertsik et al., 2012; Rees et al., 2008; Freeman et al., 2008; Shinto et al., 2016; Su et al., 2003; Khajehnasiri et al., 2015; Tayama et al., 2019; Park et al., 2015; Taheri et al., 2018; ; Yang et al., 2019; Bot et al., 2010; Mozaffari-Khosravi et al., 2013; Nemets et al., 2002; Jazayeri et al., 2008; Masoumi et al., 2016; Carney et al., 2010; Dashti-Khavidaki et al., 2014; Grenyer et al., 2007; González et al., 2011; Mischoulon et al., 2008; Marangell et al., 2003; Mischoulon et al., 2009; Ravi et al., 2016; Opiyo et al., 2018; Nahidi et al., 2012; Lucas et al., 2009; Najafabady et al., 2013; Mischoulon et al., 2014; Jiang et al., 2018)	38 parallel RCTs 1 factorial trial (20–432)	EPA (360 mg–2.2 g/day) + DHA (240 mg–2550 mg/day) (n = 30) EPA only or predominantly (1–3 g/day) (n = 9) DHA only (1–4 g/day) (n = 8)	18 monotherapy 9 continued usual antidepressant care 7 specific antidepressant 2 psychotherapy intervention 2 usual psychotherapy 1 psychoeducational leaflet 1 exercise	37 placebo 2 different dosages of the same product 2 EPA vs. DHA 1 exercise 1 active drug	22 no comorbidity 6 pregnancy/postpartum 2 overweight/obese 2 CVD 1 diabetes 1 haemodialysis 1 HIV 2 menopause 1 mixed 1 pain syndrome	+ 13 placebo ○ 23 placebo ○ 1 active ○ 1 other OTC + 2 adjunct vs. placebo ○ 1 adjunct vs. placebo + 1 lower dose ○ 1 lower dose + 2 EPA vs. DHA ○ 1 EPA vs. DHA + 1 EPA + DHA vs. DHA alone ○ 1 EPA + DHA vs. EPA alone	16 ✔✔ 7 ✔ 1 ✗ 1 ? (little detail) 14 NR
St John’s Wort (*Hypericum perforatum* L.) Bjerkenstedt et al. (2005), Philipp et al. (1999), Behnke et al. (2002), Hübner et al. (1994), Schrader et al. (1998), Wheatley (1997), Hänsgen et al. (1994), Pakseresht et al. (2012), Kasper et al. (2007), Sommer and Harrer (1994), Mannel et al. (2010), Kasper et al. (2006), Randløv et al. (2006), Rapaport et al. (2011), Papakostas et al. (2007), Gastpar et al. (2006), Brenner et al. (2000), Gastpar et al. (2005), Anghelescu et al. (2006), Uebelhack et al. (2004), Kalb et al. (2001), Lecrubier et al. (2002), Fava et al. (2005), Szegedi et al. (2005), Woelk (2000), Kasper et al. (2008), Friede et al. (2001), Hypericum Depression Trial Study Group (2002), Vorbach et al. (1994), Shelton et al. (2001), Lenoir et al. (1999), Laakman et al. (1998), Schrader (2000), Hansgen and Vesper (1996), Schmidt and Sommer (1993), Volz et al. (2000), Bergman et al. (1993)	37 parallel RCTs (30–570)	Most common dosage 900 mg/day (n = 22), other doses ranged 180 mg–1,800 mg/day	36 monotherapy 1 specific antidepressant	26 placebo 10 active drug 1 lower dose	4 comorbid conditions 33 no comorbidity	+ 16 placebo ○ 9 placebo + 4 active ○ 11 active − 1 active ○ 1 lower dose ? 1 active ? 1 placebo	31 ✔✔ 5 ✔ 1 NR
Saffron *(Crocus sativus* L.) (Noorbala et al., 2005; Akhondzadeh et al., 2022; Kolahdooz et al., 2023; Akhondzadeh et al., 2004; Abedimanesh et al., 2017; Akhondzadeh et al., 2020; Shahmansouri et al., 2014; Jelodar et al., 2018; Sahraian et al., 2016; Akhondzadeh et al., 2005; Lopresti et al., 2019; Talaei et al., 2015; Tabeshpour et al., 2017; Kashani et al., 2018; Milajerdi et al., 2018; Mazidi et al., 2016; Akhondzadeh et al., 2008)	18 parallel RCTs (30–160)	Saffron stigma (n = 10, most common 30 mg/day, (n = 7, others ranged from 15 mg to 100 mg/day) Crocin extracts (n = 3, 15–30 mg/day) Saffron petals (n = 1, 30 mg/day), mixture of stigma and petals (n = 1, 30 mg/day) Part not specified (n = 5, 30 mg/day)	14 monotherapy 3 specific antidepressant 1 usual antidepressant 1 other OTC (curcumin)	11 placebo 6 active drug 2 other OTC (different saffron products) 1 no treatment	12 no comorbidities 3 postpartum 2 CVD 1 menopause 1 type 2 diabetes	+ 8 placebo ○ 3 placebo ? 1 placebo ○ 6 active ○ 2 other OTC ○ 1 high vs. low dose ○ 1 no treatment	13 ✔✔ 2 ✔ 4 NR
Probiotics (Baião et al., 2023; Pinto-Sanchez et al., 2017; Akkasheh et al., 2016; Schaub et al., 2022; Rudzki et al., 2019; Browne et al., 2021; Ghorbani et al., 2018; Arifdjanova et al., 2021; Tian et al., 2022; Romijn et al., 2017; Rahimlou et al., 2022; Lee et al., 2021; Freijy et al., 2023; Kazemi et al., 2019; Mahboobi et al., 2022; Chahwan et al., 2019)	16 parallel RCTs (40–156)	13 multispecies 3 single species	9 monotherapy 3 specific antidepressant 3 usual antidepressant 2 usual non-pharmacological 1 high-prebiotic diet	16 placebo 1 high-prebiotic diet 1 prebiotics	11 no comorbidity 1 IBS 1 mixed 1 MS 1 pregnancy 1 obesity	+ 9 placebo ○ 6 placebo ○ 1 other OTC ? 1 placebo ○ 1 + high prebiotic diet vs. placebo	8 ✔✔ 2 ✔ 6 NR
Vitamin D (Omidian et al., 2019; Yosaee et al., 2020; Rouhi et al., 2018; Irandoust and Taheri, 2017; Far et al., 2018; Kaviani et al., 2022; Kumar et al., 2022; Alghamdi et al., 2020; Penckofer et al., 2022; Zhu et al., 2020; Zhang et al., 2018; Hansen et al., 2019; Kaplan et al., 2015; Khoraminya et al., 2013)	13 parallel RCTs 1 2×2 factorial RCT (42–158)	Daily dosages (n = 8) ranged from 1,000 to 4,000 IU/day. Weekly dosages (n = 4 trials) ranged from 50,000–100,000 IU 60,000 IU once every 5 days (n = 1) 50,000 IU dose biweekly (n = 1)	5 monotherapy 1 exercise 3 CBT and/or antidepressant 3 usual prescribed medication 2 specific drug	9 placebo 3 no treatment 1 different dose of same product 1 exercise only and no treatment (5 arm) 2 other OTC	5 no comorbidity 3 vitamin D deficiency 1 type 2 diabetes 1 T2DM + vitamin D deficiency 2 overweight/obese 1 recurrent TB infection 1 postpartum	+ 6 placebo ○ 3 placebo ○ 1 lower dose ○ 2 other OTC − 1 other OTC + 2 no treatment ○ 1 no treatment ? 1 no treatment	6 ✔✔ 1 ✔ 7 NR

**TABLE 3:** Products with emerging evidence.

Product	N studies (sample size range)	Dose	N as adjunct to other depression treatment	Comparator	N comorbid condition	Effect on depression	Safety concern
Folic acid (Papakostas et al., 2012; Coppen and Bailey, 2000; Venkatasubramanian et al., 2013; Sepehrmanesh et al., 2016; Baflo, 2009; Resler et al., 2008; Papakostas et al., 2014; Bedson et al., 2014)	6 parallel RCTs 2 sequential parallel RCTs (27–475)	0.5–15 mg/day	5 specific antidepressant 3 usual antidepressant	6 placebo 1 no treatment 1 different dose of the same product	8 no comorbidities	+ 5 placebo ○ 2 placebo − 1 no treatment + 1 lower dose	6 ✔✔ 2 NR
Lavender (*Lavandula angustifolia* Mill.) (Nikfarjam et al., 2013; Nikfarjam et al., 2017; Akhondzadeh et al., 2003; Araj-Khodaei et al., 2020; Kamalifard et al., 2017; Bazrafshan et al., 2022)	6 parallel RCTs (48–156)	Tea (n = 3, two cups of 1.5–5 g/day) Capsules (n = 2, 2 g/day) Tincture (60 drops/day 1:5)	3 monotherapy 3 specific antidepressant	3 placebo 3 other OTC 2 no treatment 2 active drug	4 no comorbid conditions 2 postmenopausal	+ 3 placebo ○ 1 active − 1 active ○ 3 other OTC + 2 no treatment	3 ✔✔ 3 NR
Melatonin (Dolberg et al., 1998; Fava et al., 2012; Serfaty et al., 2010; Mirsepassi et al., 2016; Akhondzadeh et al., 2022)	5 parallel RCTs (24–142)	3–6 mg/day	3 specific antidepressant 1 usual antidepressant 1 weight reduction diet	4 placebo 1 no treatment 1 active drug	4 no comorbidities 1 overweight/obesity	+ 2 placebo ○ 2 placebo ○ 1 active + 1 no treatment	4 ✔✔ 1 ?
Zinc (Yosaee et al., 2020; Ranjbar et al., 2013; Nowak et al., 2003; Nazarinasab et al., 2017)	3 parallel 1 2 × 2 factorial (20–140)	25–30 mg/day	1 monotherapy 3 specific antidepressant	4 placebo 1 other OTC	2 no comorbidities 1 overweight/obese 1 mixed	+ 4 placebo + 1 other OTC	4 NR
Magnesium (Mahboobi et al., 2022; Tarleton et al., 2017; Afsharfar et al., 2021; Rajizadeh et al., 2017)	3 parallel 1 crossover (46–126)	500 mg/day magnesium oxide (n = 2) 500–2,000 mg/day magnesium chloride (n = 2)	3 monotherapy 1 usual treatment	3 placebo 1 no treatment	2 no comorbidities 1 magnesium deficiency 1 obesity	+ 2 placebo ○ 1 placebo + 1 no treatment	2 ✔✔ 2 NR
Curcumin (extract from *Curcuma longa* L.) (Lopresti and Drummond, 2017; Lopresti et al., 2015; Yu et al., 2015)	3 parallel RCTs (56–123)	500–2,000 mg/day	1 monotherapy 2 specific antidepressant	3 placebo 1 low dose	2 no comorbidities 1 mixed conditions	+ 1 placebo ○ 1 placebo ○ 1 high vs. low dose ? 1 placebo	1 ✔✔ 1 ✔ 1 NR
Rhodiola (*Rhodiola rosea* L.) (Mao et al., 2015; Darbinyan et al., 2007; Gao et al., 2020)	3 parallel three-arm RCTs (57–100)	340 mg–1,360 mg/day	2 monotherapy 1 specific antidepressant	2 placebo 2 high vs. low dose 1 active drug	3 no comorbidities	+ 4 placebo − 1 placebo ○ 1 active ○ 2 high vs. low dose	3 ✔✔
Lemon balm *(Melissa officinalis* L.) (Araj-Khodaei et al., 2020; Solberg, 2011; Safari et al., 2023)	3 parallel RCTs (50–66)	600 mg–2 g/day	2 monotherapy 1 another product	2 placebo 1 no treatment 1 active drug 1 other OTC	2 no comorbidity 1 type 2 diabetes	+ 1 placebo + 1 adjunct vs. placebo ○ 1 active drug ○ 1 other OTC ○ 1 no treatment	2 ✔✔ 1 NR
Tryptophan (Levitan et al., 2000; Thomson et al., 1982; Kline and Shah, 1974)	3 parallel RCTs (34–115)	1–6 g/day	2 specific antidepressant 1 monotherapy	2 placebo 1 no treatment 2 active drug	3 no comorbidities	+ 2 placebo + 1 adjunct to active vs. placebo ○ 2 active + 1 no treatment	2 ✔✔ 1 NR
Echium *(Echium amoenum* Fisch. & C.A. Mey) (Sayyah et al., 2006; Najafabady et al., 2019)	2 parallel RCTs (35–72)	125–500 mg/day	2 monotherapy	1 placebo 1 active drug	2 no comorbidity	○ 1 placebo + 1 active	1 ✔✔ 1 NR
Bitter orange *(Citrus x aurantium* L.) (Kamalifard et al., 2017; Zare et al., 2019)	2 parallel (60–156)	1–2 g/day	1 monotherapy 1 specific antidepressant	2 placebo 1 other OTC	1 postpartum 1 menopause	+ 2 placebo ○ 1 other OTC	1 ✔✔ 1 NR
*Nepeta menthoides* Boiss. & Buhse (Kolouri et al., 2016; Firoozabadi et al., 2015)	2 parallel (43–72)	800 mg/day	1 monotherapy 1 usual antidepressant	1 active drug 1 no treatment 1 other OTC	2 no comorbidity	+ 1 active drug ○ 1 other OTC + 1 no treatment	1 ✔✔ 1 NR
Cinnamon *(Cinnamomum* spp.) (Shabanian et al., 2018; Ghaffari et al., 2020)	2 parallel (50–140)	500 mg capsules Drops, dosage not reported	1 specific antidepressant 1 usual antidepressant	2 placebo 2 other OTC	1 no comorbidity 1 low libido	+ 1 placebo ○ 1 placebo ○ 2 other OTC	1 ✔✔ 1 NR
Chamomile *(Matricaria recutita* L.) (Bazrafshan et al., 2022; Kermanian et al., 2018)	2 parallel (74–96)	2 g tea bag twice a day or 2.5 g tea bag three times per day	2 monotherapy	1 no treatment 2 other OTC	1 menopause 1 Type 2 diabetes	+ 1 placebo + 1 other OTC ○ 1 other OTC	1 ✔✔ 1 NR
SAMe (Sarris et al., 2018; Bambling et al., 2015)	2 parallel RCTs (36-107)	800–3,200 mg/day	2 usual antidepressants	1 placebo 1 different dose of same product	1 comorbid psychiatric illness 1 mixed	○ 1 placebo ○ 1 lower dose	2 ✔✔
Vitamin D + calcium (Amini et al., 2022; Jorde et al., 2008)	1 parallel RCT 1 factorial RCT (81–441)	Vitamin D (50,000 IU fortnightly or 40,000 IU/week) + calcium (500 mg/day)	2 monotherapy	2 placebo 1 different dose of same product 1 other OTC	1 postpartum 1 comorbid conditions	+ 1 placebo − 1 vitamin D alone ? 1 placebo ? 1 lower dose	1 ✔✔ 1 NR
Vitamin C (Khajehnasiri et al., 2015; Sahraian et al., 2015)	1 parallel RCT 1 factorial RCT (68–136)	500–1,000 mg/day	1 monotherapy 1 specific antidepressant	2 placebo 1 other OTC	2 no comorbidity	○ 2 placebo ○ 1 active	1 ✔✔ 1 NR
Prebiotics (Kazemi et al., 2019; Vaghef-Mehrabany et al., 2021)	2 parallel RCTs (62–110)	5 g/day galactooligosaccharides Inulin 10 g/day	2 usual antidepressant	2 placebo 1 other OTC	1 no comorbidity 1 obesity	○ 2 placebo ○ other OTC	2 ✔

**TABLE 4:** Single products evaluated in single trials.

Product	Dose, form, and adjunct	Sample size Diagnosis or symptoms Comorbid condition	Effect on depression	Safety concern
4G-beta-D-galactosucrose (Tarutani et al., 2022)	7 g/day Syrup Usual antidepressant + dietary guidance	22 Diagnosis None	- Placebo	NR
*Asperugo procumbens* L. (Zarghami et al., 2018)	400 mg/day Capsules Monotherapy	30 Diagnosis Mixed	- Fluoxetine	✔✔
Aspirin (Berk et al., 2020)	100 mg/day Adjunct to usual antidepressant/psychotherapy	130 Diagnosis None	○ Placebo	✔✔
Astaxanthin (Hayashi et al., 2020)	12 mg/day Jellies (2 mg containing 1% astaxanthin powder) Monotherapy	60 Symptoms None	○ Placebo	✔✔
Basil (*Ocimum basilicum* L.) (Karimi et al., 2021)	500 mg/day Capsules Monotherapy	76 Symptoms Postmenopausal women	+ Placebo	NR
*Chlorella vulgaris* (Panahi et al., 2015)	1,800 mg/day Microalgae tablets Adjunct to usual antidepressant	125 Diagnosis None	+ No treatment	✔✔
Chromium picolinate (Davidson et al., 2003)	600 mcg/day Capsules Monotherapy	16 Diagnosis None	+ Placebo	✔✔
*Cuscuta planiflora* Ten. (Firoozabadi et al., 2015)	2 g/day Capsules Adjunct to usual antidepressant	43 Diagnosis None	+ No treatment ○ Other OTC	NR
Fenugreek (Torkestani et al., 2013)	6 g/day Seeds Monotherapy	60 Symptoms Menopause	? placebo	NR
Flavonoid-rich orange juice (Park et al., 2020)	190 mL twice a day Juice Monotherapy	40 Symptoms None	+ Placebo	NR
Ginger (*Zingiber officinale* Roscoe) (Shabanian et al., 2018)	Dose NR Drops Adjunct to antidepressant (if taking)	140 Symptoms Loss of libido	○ Placebo	NR
Green tea (*Camellia sinensis* (L.) Kuntze) (Manshadi Seyed Ali et al., 2021)	800 mg/day Capsules Monotherapy	50 Diagnosis HIV patients receiving antiretroviral therapy	+ Placebo	✔✔
Inositol (Levine et al., 1999)	12 g/day 1 tsp powder in juice or tea four times a day Adjunct to SSRI	36 Diagnosis None	○ Placebo - Imipramine	✔✔
L-carnitine (Zanardi and Smeraldi, 2006)	1,000 mg/day Tablets Monotherapy	204 Diagnosis (dysthymia) None	○ Amisulpride	✔✔
L-tyrosine (Gelenberg et al., 1990)	100 mg/kg/day Tablets Monotherapy	65 Diagnosis None	○ Placebo	✔✔
Lotus seeds (*Nelumbinis* semen) (Ye et al., 2022)	2.4 g and 4.8 g/day Capsules Monotherapy	46 Symptoms None	+ Placebo	✔✔
*Nigella sativa* L. (Rafiee et al., 2022)	100 mg oil/day Capsules Adjunct to usual sertraline	54 Diagnosis None	+ Placebo	✔
Rose (*Rosa x damascene* Herrm.) (Shabanian et al., 2018)	Dose NR Drops Adjunct to antidepressant (if taking)	140 Symptoms Loss of libido	○ Placebo	NR
Rosemary (*Rosmarinus officinalis* L.) (Azizi et al., 2022)	700 mg/day Capsules Adjunct to SSRI	59 Diagnosis None	+ Placebo	✔
Soy isoflavones (De Sousa-Muñoz and Filizola, 2009)	176 mg/day Capsules Monotherapy	84 Symptoms Menopause	○ Placebo	✔
Sumac (*Rhus coriaria* L.) (Hariri et al., 2020)	3000 mg/day Capsules Restricted calorie diet	62 Diagnosis Overweight/obese	○ Placebo	NR
Ulva (*Ulva Lactuca* L.) (Allaert et al., 2018)	390–650 mg/day depending on body weight Capsules Monotherapy	90 Symptoms None	+ Placebo	✔✔
Vitamin B6 (Adams et al., 1973)	40 mg/day Tablets Monotherapy	32 Symptoms None	? Placebo (within groups only)	NR

**TABLE 5:** Summary of mixed products.

Product (reference) Dose Adjunct	Sample size Dx/Sx depression Comorbid conditions?	Effect on depression	Safety concerns
Amino acids + B12 (Fontan, 1991) Dose NR, 2 tablets daily Adjunct to amitriptyline	60 Symptoms None	+ Placebo	NR
200 mg SAMe (200 mg), B1 (3 mg), B2 (3.4 mg), B3 (25 mg), and B6 (2 mg) + B12 (1 μg) (Djokic et al., 2017) 1 capsule daily Monotherapy	60 Diagnosis None	+ Placebo	NR
SAMe (800 mg/day), folic acid (500 mcg/day) and co-factor vitamin B12 (200 mcg/day), omega-3 fatty acid concentrate (EPA-esters 1,000 mg/day; DHA-esters 656 mg/day), 5-HTP (200 mg/day), zinc picolinate (30 mg elemental/day); vitamin B6 (100 mg/day), vitamin C (60 mg/day), magnesium (amino acid chelate, elemental 40 mg/day), and vitamin E (40 IU/day) (Sarris et al., 2019) 2 capsules and 2 tablets twice a day Adjunct to antidepressant	158 Diagnosis Mixed	○ Placebo	✔✔
Probiositive: SAMe (200 mg) + probiotics (*Lactobacillus helveticus* Rosell^®^− 52, *Bifidobacterium longum* Rosell^®^− 175 (3 × 10^9^ CFU)) + magnesium oxide (93.30 mg), and B6 (1.70 mg) (Ullah et al., 2022) 1 tablet daily Monotherapy	65 Symptoms None	+ Placebo	NR
SAMe (200 mg) and *L. plantarum* (HEAL9 1 × 10^9^ CFU) (Saccarello et al., 2020) 1 tablet daily Monotherapy	90 Diagnosis None	+ Placebo	✔✔
Tryptophan (100 mg) and vitamin B6 (4 mg) + nicotinamide (4 mg) (Tsujita et al., 2019) 2 capsules daily Monotherapy	30 Symptoms None	+ Placebo	NR
Enlyte: folic acid (1 mg), folinic acid (2.5 mg), L-methylfolate magnesium (7 mg), thiamine pyrophosphate (25 mcg), flavin adenine dinucleotide (25 mcg), pyridoxal 5-phosphate (25 mcg), adenosylcobalamin (50 mcg), thiamine pyrophosphate (25 mcg), NADH (25 mcg), trimethyl glycine (500 mcg), AminoFerr (1.5 mg), magnesium ascorbate (24 mg), zinc ascorbate (1 mg), and L-threonic acid magnesium (1 mg) + Sharp PS Gold (20 mg) (Mech and Farah, 2016) Capsules, N not reported Monotherapy	330 Diagnosis None	+ Placebo	✔✔
1412 mg omega-3 fatty acids, 30 μg selenium, 400 μg folic acid, and 20 μg vitamin D3 plus 100 mg calcium (Bot et al., 2019) 2 pills per day Adjunct to behavioural activation and monotherapy (2 × 2 factorial trial)	1025 Symptoms Overweight/obesity	○ Placebo No interaction with BA (factorial trial)	✔✔
Vitamin B complex; 20 mg B6, 500 mg B12, 50 mg thiamine, 20 mg riboflavin, 400 mg folate, 50 mg delta-pantothenic acid, 300 mg biotin, 300 mg niacin, and 20 mg intrinsic factor (Kaplan et al., 2015) 1 capsule per day Monotherapy	56 Symptoms None	○ Vitamin D ○ Broad spectrum multivitamin	✔✔
Broad spectrum multivitamin: 384 mg vitamin A, 8 mg B6, 200 mcg B12, 133.2 mg vitamin C, 320 m IU vitamin D, 53.6 mg vitamin E, 4 mg thiamine, 3.2 mg riboflavin, 320 mg folate, 4.8 mg delta-pantothenic acid, 240 mg biotin, 20 mg niacin, 138.8 mcg chromium, 1.6 mg copper, 45.2 mcg iodine, 3.2 mg iron, 293.3 mg calcium, 133.2 mg magnesium, 2.0 mg manganese, 186.8 mg phosphorus, 53.2 mg potassium, and 45.2 mcg selenium + 10.8 mg zinc (Kaplan et al., 2015) 4 capsules per day Monotherapy	56 Symptoms None	○ Vitamin D ○ Vitamin B complex	✔✔
Omega-3 (180 mg EPA and 120 mg DHA) + vitamin C (250 mg) (Khajehnasiri et al., 2015) 1 capsule of each twice a day Monotherapy	136 Symptoms None	○ Placebo	NR
St John’s Wort (1.8 g) + kava (2.66 g) (Sarris et al., 2009) 1 tablet each three times a day monotherapy	28 Diagnosis None	? Placebo (conflicting results at different periods of the crossover trial)	✔✔
Neurapas: St John’s Wort (80 mg), passionflower (40 mg), valerian (40 mg), corydalis (40 mg), and Californian poppy (40 mg) (Ditzier et al., 1994) 2 tablets three times a day	60 Symptoms None	+ Placebo	✔
Saffron (64 mg), cinnamon (357 mg), and St John’s Wort (857 mg) in 10 mL (Adalat et al., 2019) Syrup 10 mL twice a day	52 Symptoms Multiple sclerosis	+ Placebo	✔✔
Equal parts of Brahmi, Shankhpushpi, Malkangni, and Jatamansi; 125 mg total (Bhargava and Khan, 2012) 500 mg twice a day Monotherapy	90 Diagnosis None	○ Imipramine ○ Sertraline	✔✔
Lavender (*Lavandula angustifolia* L. flowers 15 g in 400 mL water) and dodder (*Cuscuta chinensis* Lam. seeds 15 g in 400 mL water) (Firoozeei et al., 2020) Syrup 2 × 5 mL/day + placebo tablet Monotherapy	56 Diagnosis Anxiety	○ Citalopram (+ placebo syrup)	✔✔
Lemon balm (*Melissa officinalis L.* L., 1000 mg) + *Nepeta menthoides* Boiss. & Buhse (400 mg) capsules (Ranjbar et al., 2018) 3 × 500 mg capsules daily Monotherapy	54 Symptoms Anxiety and insomnia	+ Placebo	? (no detail by group)
Aphrodite: ginger (12.27 mg), saffron (3 mg), cinnamon (11 mg), thistle (14 mg) (all Latin names not reported), and *Tribulus terrestris* L. (40 mg) (Shahmoradi et al., 2023) 2 tablets per day Adjunct to usual sertraline	54 Diagnosis SSRI-induced sexual dysfunction	+ Placebo	NR

### Summary of included studies

Trials were most commonly carried out in Iran (n = 78), followed by Germany (N = 28), the United States (n = 27), Australia (n = 13), the United Kingdom (n = 9), and China (n = 6). Other countries had five or fewer studies. The vast majority of studies were parallel trials (n = 194), with three factorial trials and four crossover trials. Sample sizes varied widely, with a median size of 70 participants (IQR, 46–123) recruited and a median of 62 participants analysed (IQR 42–106). Most studies were double-blind (n = 187), six were triple-blind, six were single-blind, six were open-label, and four did not report blinding (based on the study details, three were likely to be open-label and one likely single-blind).

In 150 trials, participants were required to have a diagnosis of depression at baseline. In 58 trials, a symptom scale cutoff was used to determine inclusion, with participants needing to meet a minimum threshold. In one study, a diagnosis was implied but not clearly stated, but a scale cutoff was also used. All studies used continuous depression scales as primary or secondary outcomes. Most scales were clinician-rated and tended to be summarised continuously rather than as response or remission; the most common were the Hamilton Depression Rating Scale (106 primary and 13 secondary outcomes), the Montgomery–Åsberg Depression Rating Scale (17 primary and 10 secondary outcomes), and the Clinical Global Impression Scale (8 primary outcomes and 40 secondary outcomes). The self-reported Beck Depression Inventory was also commonly used (61 primary and 33 secondary outcomes). Other scales were used in fewer than 10 studies.

Dietary supplements were evaluated in 114 trials, HMPs in 94 trials, and single chemical medicines in 1 trial. No studies evaluated homoeopathic products. More herbal medical product trials included people with a diagnosis of depression than dietary supplement trials (74/94, 79% vs. 75/114, 66%). Most studies (n = 156) had two trial arms, 42 had three arms, 10 had four, and 1 trial had five arms. Trials with multiple arms could include comparisons with lifestyle changes, other OTC products, prescribed drugs, and placebos. Dietary supplements were more frequently compared to placebo (n = 98/114, 86%) than HMPs (n = 64/94, 67%), whilst HMPs were more frequently compared to prescription drugs (n = 32/94, 32%) than dietary supplements (n = 6/114, 5%).

Dietary supplements (n = 67/114, 59%) were also more likely to be evaluated as adjuncts to other depression treatments (most commonly antidepressants) compared to HMPs (21/94, 20%). Very few studies evaluated products versus psychotherapy-based treatments (n = 4) or as adjuncts to psychotherapy-based treatments (n = 11). Seventeen studies contained comparisons between different OTC products. HMPs were evaluated in people with comorbid health conditions in 22/94 (23%) studies and 32/114 (28%) trials of dietary supplements. Regarding pregnancy status, 4/94 studies evaluated HMPs during the postpartum period, and 9/114 dietary supplements were evaluated during pregnancy or postpartum.

The studies show a general increase in the number of trials evaluating dietary supplements for depression since 1973, peaking in 2019, with an average of 3.2 trials per year. The number of trials evaluating HMPs remained steady, with smaller peaks and troughs and an average of 2.7 trials published per year since 1993. This suggests a sustained interest in OTC products for depression over time.

### Products with substantive evidence (>10 trials)

We found that trial evidence was concentrated on a small number of products, namely, omega-3s (n = 39, median sample size: 65), St John’s Wort (n = 38, median sample size: 150), saffron (n = 18, median sample size: 50), probiotics (n = 18, median sample size: 71), and vitamin D (n = 14, median sample size: 67). Within these categories, however, there was considerable variation in the specific products, extracts, and dosages tested (see Table 1 for further details).

The vast majority of omega-3 trials (n = 37/39) compared the supplement to a placebo, with a small number of studies assessing different dosages or comparing eicosapentaenoic acid (EPA) to docosahexaenoic acid (DHA). A total of 26 trials were conducted in people with a diagnosis of depression, 12 in those with depressive symptoms, and 1 was unclear. In 11 studies, participants had a range of comorbidities, six of which involved pregnancy/postpartum, and in 16 studies, they were evaluated as an adjunct to antidepressant treatment. On the basis of vote counting, the effects on depression were more frequently null compared to placebo than significantly different (23 vs. 13), with mixed results for other comparisons.

The most commonly studied herbal medical product was St John’s Wort (*Hypericum perforatum*), which was only evaluated in trials involving people with a depression diagnosis. It was given mainly as a monotherapy to people with no comorbidities. St John’s Wort was compared to a placebo in 26 trials, to an active drug in 10 trials, and to a lower dose in one trial. Using vote counting, the evidence mostly favoured St John’s Wort, with more positive trials compared to placebo (16 positive vs. 9 null) and producing similar or better effects compared to prescription antidepressants (11 similar effects, four positive effects, and one worse effect).

Saffron products were mostly evaluated as a monotherapy compared to placebo. Fourteen trials included participants with a diagnosis of depression, and four focussed on participants with depressive symptoms. A range of extracts were evaluated, including stigma (n = 10), crocin (n = 3), petals (n = 1), mix (n = 1), and unspecified types (n = 5). Saffron was evaluated across a wider range of comorbid conditions than St John’s Wort, including cardiovascular disease, postpartum, menopause, and type 2 diabetes. Generally, positive effects were found compared to placebo (eight trials reported positive effects, and three trials reported null findings), and comparisons with prescription medications indicated similar effects (n = 6) as did comparisons between different saffron products (n = 2).

Probiotics were evaluated mainly as multispecies products (n = 13/16), with three evaluating single strains, compared to a placebo (n = 16). Equal numbers of studies were carried out in people with a diagnosis (n = 8) or symptoms (n = 8) of depression. In five studies, people with a range of comorbidities were included, and in six studies, they were evaluated as an adjunct to antidepressant therapy (n = 7/16). Results by vote counting favoured probiotics vs. placebo (nine positive vs. six null), with similar results to other OTC products and a combination of probiotics plus a high prebiotic diet vs. placebo.

Vitamin D trials encompassed a variety of dosing regimens (daily to weekly) and levels (1,000–100,000 IU). Nine trials were conducted in people with a depression diagnosis, and six trials were conducted in people with depressive symptoms. In most cases, vitamin D was evaluated as an adjunct to prescribed medication and/or Cognitive behavioural therapy (n = 8/14); it was most commonly compared to placebo (n = 9/14) and often assessed in people with comorbid conditions (n = 9/14, mostly vitamin D deficiency). Comparisons with placebo mainly favoured the vitamin D group (six positive vs. three null), but other types of comparisons showed mixed results.

### Products with emerging evidence (2–9 trials)

Eighteen products were tested in 2–7 trials per product (see Table 2). Out of these, folic acid (n = 8), lavender (n = 6), zinc (n = 4), tryptophan (n = 3), rhodiola (n = 3), and lemon balm (n = 3) were the most promising. Doses and preparations in these studies were variable—for example, folic acid doses ranged from 0.5 mg to 15 mg per day, whilst lavender was evaluated as a tea, capsules, and tincture. Among the products evaluated in only two trials, positive effects were found for bitter orange, *Nepeta menthoides* Boiss. & Buhse, and chamomile tea. The vast majority of these trials involved people without comorbidities, with the exceptions being bitter orange (evaluated in one postpartum and one menopause trial) and chamomile tea (evaluated in one menopause and one type 2 diabetes trial). Mixed results were found for melatonin (n = 5), magnesium (n = 4), curcumin (n = 3), cinnamon (n = 2), Echium (n = 2), vitamin C (n = 2), and vitamin D plus calcium (n = 2). Trials found null results for SAMe (n = 2) and prebiotics (galactooligosaccharides or inulin) (n = 2).

### Products with single trials

Forty-one products were evaluated in one trial each. Nineteen products were of a single substance (13 HMPs, 9 dietary supplements, and 1 chemical (aspirin); see Table 4), and 18 contained mixes of different dietary supplements (n = 11) or mixed HMPs (n = 7). Mixed dietary supplements frequently contained SAMe (n = 4) and various B vitamins in addition to other vitamins and minerals. Mixed HMPs tended to contain different combinations of HMPs that were also evaluated alone, with St John’s Wort included most often (n = 3). The compositions of the mixed products are listed in Table 5.

For single-substance products, positive effects compared to placebo were found for rosemary, green tea, lotus seeds, ulva, basil, chromium, *Nigella sativa* L., and flavonoid-rich orange juice. Compared to no treatment, Cuscuta and Chlorella showed positive effects. Null effects were found for aspirin, inositol, L-tyrosine, and astaxanthin compared to placebo. L-carnitine showed non-inferiority to amisulpride for depressive symptoms. Inositol showed a tendency to be less effective than imipramine, 4G-beta-D-galactosucrose was less effective than placebo, and asperugo was significantly less effective than fluoxetine. No effects compared to placebo were found for soy extract, sumac, rose, and ginger. Two trials (of fenugreek and vitamin B6) only reported within-group analyses, so their effectiveness was not classified.

Ten mixed products showed significant effects on depressive symptoms, compared to three that showed no effect and one with unclear results (due to conflicting results at different periods of the crossover trial). One supplement showed no interaction with behavioural activation in a 2 × 2 factorial trial, whilst a three-arm trial showed no differences between vitamin D, a vitamin B complex, and a broad-spectrum multivitamin on depressive symptoms. Two trials found effects similar to prescription antidepressants for an Ayurvedic formula and lavender–dodder syrup.

### Safety evidence

Most trials (n = 145/209, 69%) reported adverse events (AEs) in sufficient detail to assess safety concerns, but a substantial portion (n = 64/209, 31%) either did not report this or reported in insufficient detail, such as failing to report by trial arm. There were no noticeable trends in the number of trials reporting AEs over time, but dietary supplement trials were less likely to report AE data (44/114, 39%) compared to herbal medical product trials (20/94, 22%). Safety concerns for each product are reported in Tables 2–5.

Of the trials that reported AEs, 123/145 (85%) reported no safety concerns (e.g., similar numbers of AEs between groups), and 21 trials found higher rates of mild AEs in the product group. Higher rates of mild AEs were generally found in products with more substantive evidence, whilst those with emerging evidence or single trials generally reported no safety concerns or did not report AE data. Only one omega-3 trial reported some safety concerns—higher rates of serious adverse events in the EPA group than that in the placebo group, with unclear relatedness (Lesperance, 2011). The proportion of studies reporting increased mild AEs in the intervention group was similar whether products were administered alone (14/121, 12%) or as adjunct therapy (7/89, 8%) (notably, one study included one adjunct and one monotherapy arm, so it is included in both groups). Mixed products showed similar rates of no safety concerns reported (10/19, 53%) to single products (113/190, 59%), and similar proportions did not report on AEs (6/19, 32% vs. 55/190, 29%).

### Health economic evidence

We found one trial which assessed the economic impact of folic acid on depression symptoms over 25 weeks. From a National Health Service perspective, folic acid was not more effective than a placebo but was slightly cheaper, with no significant differences in costs or resource use.

### Ongoing trials

Forty-seven ongoing trials were found with a protocol or registry entry but no publication, of which 34 evaluated dietary supplements and 13 evaluated HMPs (see Supplementary Material). Most were probiotics (n = 17/34), with four vitamin D trials and three omega-3 trials. A range of HMPs were evaluated. Thirty trials reported evaluating depression alone, with 17 also including people with a range of comorbid conditions, including 6 trials for anxiety/insomnia/stress. Four trials were terminated early—two due to COVID-19, one due to recruitment difficulties, and in one, the reason was not reported.

Most products reflect those that have been evaluated previously; however, new products with no current trials located in this review for depression included resveratrol, hydrangea, cordyceps, dill, 5-HTP, creatine, ashwagandha, *Bacopa monnieri* (L.) Wettst. *+ Nardostachys jatamansi* (D.Don) DC., three multi-nutrient products, cannabidiol*, Platycodon grandiflorum* extract + *Poncirus trifoliata* (likely to be *Platycodon grandiflorus* (Jacq.) A. DC. + *Poncirus trifoliata* (L.) Raf.), and Harmal powder (no further details). There were no noticeable emerging trends in the number of trials per product.

## Discussion

This review found a total of 209 trials assessing OTC products in people with depressive symptoms or a depression diagnosis. Most products evaluated were dietary supplements or herbal medical products. Only one single chemical medication was found (aspirin), and no homeopathic products were found. We identified notable gaps in the evidence:

• Although a wide range of products have been evaluated, for many of these, we found only a small number of trials, and further replication of results is needed. Ongoing studies currently follow this trend and either evaluate products already with a substantive evidence base or completely new products.
• Evidence is concentrated on five key products (omega-3s, St John’s Wort, saffron, vitamin D, and probiotics). However, these still include wide variations in dose and preparation.
• Many mixed products are available on the market (or are likely to be used simultaneously), but few trials assessed mixed products.
• Few herbal teas have been evaluated, with most herbal medical product trials focussing on capsule formulations.
• Most trials used small to medium sample sizes; more definitive trials are needed as smaller antidepressant trials have been shown to overestimate effects (Hamza et al., 2024).
• Whilst a substantial number of trials have studied OTC products as an addition to antidepressants, few trials have evaluated the effect of OTC products alongside or compared to psychological therapies despite their increasing usage.
• Most products are evaluated against placebo rather than other OTC options, which is likely to hamper patients’ ability to make informed choices.
• A substantial number of trials did not report sufficient safety data, particularly for dietary supplements. However, in those that did, most products had a good safety profile.
• Promising products worthy of further evaluation include folic acid, lavender, zinc, tryptophan, rhodiola, lemon balm, bitter orange, *Nepeta menthoides* Boiss. & Buhse, and chamomile.
• Products with mixed evidence requiring further evaluation include melatonin, magnesium, curcumin, cinnamon, Echium, vitamin C, and vitamin D plus calcium.

Questionnaires recording the use of products for depression have found that in the US, Serbia, Mexico, and Iran, the most commonly reported HMPs were borage, chamomile, lavender, St John’s Wort, ginseng, ginkgo, orange blossom, valerian, tilia, lemon balm, and peppermint (Roy-Byrne et al., 2005; Ashraf et al., 2021; Alonso-Castro et al., 2021; Stojanović et al., 2017; Niv et al., 2010). There remain notable gaps in the evidence base regarding ginseng, ginkgo, tilia, orange blossom, and peppermint, and there are relatively few studies for products other than St John’s Wort. Borage was commonly used in only one survey in Iran; although the species name was not specified, it is likely to refer to Iranian borage (*Echium amoenum* Fisch. & C.A.Mey), which was evaluated in two studies included in this review, rather than *Borago officinalis* L. Echium showed mixed results for its effects on depression and warrants further research. Products containing mixes of herbs and/or dietary supplements were rarely evaluated. This may arise from the fact that standardisation and ensuring supply chain quality are more challenging when multiple extracts are present (Ekor, 2014). Future research should, therefore, focus on individual extracts and evaluating potential additive effects of effective herbal products using methods such as factorial trials, which were rarely used in this review. No homoeopathic products were found that were evaluated for depressive symptoms in this review; this suggests that this is not a good avenue for further research at present.

Products also rarely focused on teas (a small number of lavender and chamomile products), as opposed to capsules, even though in some studies, teas were the most common way of consuming HMPs for depression (Stojanović et al., 2017). Lemon balm (75.9%) and chamomile (60.3%) were the two most commonly used teas in a Portuguese consumer panel (Rocha et al., 2020). Herbal tea consumers closely associated herbal teas with emotions (e.g., calm and relaxation), sensory experiences (e.g., taste and appearance), and perceived effects on health and wellbeing (Rocha et al., 2020). Herbal teas are generally under-researched; a scoping review of the general health benefits of herbal teas found only 16 clinical studies and five observational studies (Poswal et al., 2019). Given their wide availability, low cost, and ability to become part of an everyday lifestyle, herbal teas warrant further clinical and observational research for their role in managing depression.

With regards to dietary supplements, there was a particular gap between the most commonly used products and the evidence base available. One study from New Zealand found that the most common dietary supplement among people with depression was a multivitamin, followed by vitamin B complex (Silvers et al., 2006), while a US study found that people with psychiatric symptoms were more likely to take melatonin compared to those without psychiatric symptoms (Niv et al., 2010). Only a small number of trials evaluated these products for depression, with few positive effects. Rates of omega-3 consumption were not significantly different in a large US survey in those with and without psychiatric symptoms (Niv et al., 2010) despite its substantive evidence base. We found few trials for multivitamins, and these had variable results. Multivitamins in particular present a challenge to evaluate due to the potential for variation between the number, type, and dose of vitamins and minerals included. Melatonin (more commonly used for insomnia) showed mixed effects on depressive symptoms and may warrant further research as a potential treatment for depression.

The use of OTC single-chemical products for depression has been evaluated in relatively few studies. This is not particularly surprising as most single-chemical medications targeting depression are prescription-only. One Swedish study found that adolescents with depressive symptoms were more likely to use OTC analgesics than their non-depressed peers (Hena et al., 2019); however, few studies have explored this relationship in adults. A single trial of aspirin (hypothesised to exert effects through anti-inflammatory mechanisms) found no effects on depressive symptoms. Few randomised trials have assessed aspirin for depression; however, a review of prospective cohort studies found that aspirin use was associated with an increased risk of depression (Kim et al., 2020). Although this may arise from confounding factors such as underlying pain or cardiovascular disease, the existing evidence suggests that aspirin is unlikely to be a promising candidate for future depression research.

Whilst a substantive evidence base was found for a small number of products, existing meta-analyses provide relatively recent conclusions regarding the level and quality of evidence available for these products. A 2019 meta-analysis of nine saffron studies found it to be effective against placebo and not significantly different from antidepressants, with studies mostly having a low or unclear risk of bias (Tóth et al., 2019). A 2017 meta-analysis of 27 studies on St John’s Wort showed similar efficacy and lower dropout rates compared to antidepressants, supported by a moderate-quality evidence base (Ng et al., 2017). Omega-3s showed significant effects in reducing depressive symptoms compared to placebo across 26 studies, with greater effects in EPA-enriched or EPA-only products (Liao et al., 2019). The evidence base for probiotics (19 trials) was generally of high quality and showed that probiotics reduce depressive versus placebo, with greater effects observed in people with major depressive disorder (Goh et al., 2019). Vitamin D (nine trials) showed limited evidence of efficacy to date (Gowda et al., 2015). There are some differences between these meta-analyses and the findings of our review. As included studies had only a small sample size (median 62), it is possible that positive but non-significant effects became significant when pooled, in contrast to our use of vote counting, which has well-established limitations. It may also reflect differences in search strategies and inclusion criteria. Our searches identified more trials than some of these reviews (Tóth et al., 2019; Liao et al., 2019; Gowda et al., 2015) despite similar inclusion criteria, suggesting that future meta-analyses should adopt a more thorough search approach. Other reviews found a large number of studies but employed wider inclusion criteria, including healthy samples (Goh et al., 2019). Generally, products with a more substantive evidence base had more trials showing some differences in mild adverse events; however, this may represent more rigorous detection methods or criteria rather than actual safety differences to products with emerging evidence. Only 10 studies in our review evaluated different doses of the same product; dose-response relationships require further study for all products.

Ongoing trials also focussed on omega-3s, probiotics, and vitamin D. Rather than conducting further trials of these products, future attention needs to be directed towards promising products with fewer trials. Products with fewer studies but with more positive than null results include folic acid, lavender, rhodiola, tryptophan, lemon balm, bitter orange, *N. menthoides* Boiss. & Buhse, chamomile, and zinc. Lavender, chamomile, and lemon balm are traditionally indicated and used in Western herbal medicine for depression, particularly with comorbid anxiety or stomach complaints (Bell Hunter, 2007; Bone, 2003). Bitter orange (*Citrus x aurantium* L.) and *N. menthoides* Boiss. & Buhse are traditionally used for psychological disorders, including depression, particularly in Iran (Eslami-Farouji and Jalili, 2024; Memariani et al., 2019). *Rhodiola rosea* L. is traditionally used across Europe and Asia for stress, fatigue, and depression (Tao et al., 2019). Most of these products show some evidence of influencing the pathophysiological mechanisms of depression. Lavender oil, rhodiola, lemon balm, *N. menthoides* Boisse. & Buhse, chamomile, folic acid, tryptophan, and zinc show anti-inflammatory activity in *in vitro* and *in vivo* studies (Dobrek and Głowacka, 2023; Zam et al., 2022; Dai et al., 2022; Liwinski and Lang, 2023; Wang et al., 2018; Nayak et al., 2019). Lavender, rhodiola, folic acid, and lemon balm modulate the activity of various neurotransmitters, whilst tryptophan is a precursor molecule for serotonin (Dobrek and Głowacka, 2023; Liwinski and Lang, 2023; Nayak et al., 2019). Bitter orange, *N. menthoides* Boiss. & Buhse, lemon balm, chamomile, tryptophan, and zinc show antioxidant effects (Dobrek and Głowacka, 2023; Dai et al., 2022; Wang et al., 2018; Nayak et al., 2019; Maksoud et al., 2021). There is also evidence that zinc, lemon balm, and rhodiola moderate HPA axis activity, whilst lavender, chamomile, and zinc showed neuroprotective activity by increasing or restoring BDNF (Dobrek and Głowacka, 2023; Dai et al., 2022; Wang et al., 2018). These potential mechanisms of effect, combined with traditional use and promising clinical evidence, suggest that further, larger trials should concentrate on these products for the treatment of depression. Despite this, few of these HMPs had registered ongoing trials (n = 1 lavender).

With 89 trials evaluating products as adjuncts alongside conventional treatment (mostly antidepressants), this represents a more realistic approach to evaluation (Solomon and Adams, 2015) and is welcomed. However, very few trials assessed the additional value of these therapies to psychological therapies. As these are considered potential first-line treatments in the UK (National Institute for Health and Care Excellence, 2022), this needs to be evaluated further. The substantive nature of the evidence base for some products also suggests that NICE guidelines should formally review these in future updates and provide clarity as to whether to recommend the use of these products for depression. Likewise, 54 trials included people with comorbidities. As depression is more common in people with long-term conditions (Gutiérrez-Rojas et al., 2020), this is heartening. Thirteen studies were also carried out in pregnant or postpartum women. Preliminary evidence indicated most products were safe, whether administered alone or alongside antidepressants. This will provide confidence to users of OTC products for depression, particularly those already taking antidepressants. However, 64 studies did not report adverse events, and reporting did not improve over time. Authors are strongly recommended to clearly report adverse events (or lack thereof) within OTC product trials, particularly as these products are most frequently used without clinical supervision. Further comprehensive analysis of adverse event data for each product is also recommended. Only one economic evaluation was found; further work on whether OTC products could provide savings in healthcare resource use for depression is needed.

Strengths of our review include the fact that we located a much larger number of trials than a previous review—focussing on products for all ages and for anxiety and insomnia (n = 76) (Kamat et al., 2023)—and more than some of the individual product meta-analyses discussed above, demonstrating the value of a scoping review methodology and a thorough approach to searching and follow up. We also summarised ongoing trials. We brought together multiple available products rather than focussing on a single product to provide an overall map of the evidence. We focussed on samples where participants had baseline depression symptoms or a diagnosis rather than those that examined depressive symptoms in non-depressed samples.

However, there were some key limitations to our review. Despite attempts to search exhaustively, some trials did not use product class keywords that were used in our searches, and we were unable to search each potential product name. It is, therefore, likely that some relevant studies were missed—for example, a meta-analysis of St John’s Wort (Ng et al., 2017) located more comparisons with SSRIs than those found in our review, and two potentially eligible trials of aspirin were found in another review (Dominiak et al., 2022). Future studies need to report product class, descriptors and names. Searches were also only carried out up to 2022. Follow-up of trial protocols and conference abstracts detected 16 further studies, some of which were published after the search dates. However, trial registry entries were not always up to date, and sometimes, a lack of detail limited further searching. Likewise, multiple publications from the same studies were not always clearly labelled. Only 10% of studies were screened by two authors, which may have introduced bias. In our protocol, we originally planned to have two reviewers screening all titles, abstracts, and full texts and to perform citation tracking and reference list screening of included studies; however, the large volume of studies identified and our limited resources precluded this. Future reviews should use a higher proportion of dual screening or have all studies dual-screened. Although we included non-English literature, we did not search using multi-language terms, and some studies were translated using Google Lens, which may not provide fully accurate details for data extraction. Future research should also consider including traditional Chinese medicine approaches to provide a more global perspective.

As the review was intended to be descriptive, we used simple vote counting to summarise the study’s effectiveness. This could be difficult to classify when different results occurred in multiple measures of depression or at different timepoints, and we did not account for sample size and, therefore, study power. We also did not explore the quality of statistical analyses or the use of intention-to-treat vs. per-protocol analysis, which were often poorly described and inconsistently labelled across studies. Results are, therefore, less precise than a meta-analysis or narrative review with consideration of study quality, and as such, they should be viewed as preliminary and not definitive. Safety data were variably reported and were not always tested for significance between adverse event incidences between groups; therefore, we took a cautious approach, and imbalances of more than a few between groups were classed as differences. As almost no serious adverse events were reported in trials; this is unlikely to affect review safety conclusions substantially. Including such a large number of products and trials necessarily resulted in some loss of nuance regarding dosage and preparation type.

## Conclusion

The largest volume of evidence exists for omega-3s, St John’s Wort, saffron, probiotics, and vitamin D, all of which are relatively established products. However, a multitude of other products are promising, including folic acid, lavender, zinc, tryptophan, rhodiola, and lemon balm. Likewise, several products have mixed evidence, requiring further study such as melatonin, magnesium, curcumin, cinnamon, Echium, vitamin C, and vitamin D plus calcium. Among these, chamomile, lavender, lemon balm, and Echium represent commonly used products and should be prioritised for further research into their safety and effectiveness.

Most studies reporting on safety presented no safety issues, with a small number showing differences in mild adverse effects. One study on an omega-3 product reported a higher incidence of serious adverse events in the product group. Safety data were consistent whether the products were used alone or as adjuncts to antidepressants. However, trial authors should ensure that such information is reported completely and clearly in papers. There is a need for further evaluation of HMPs as adjuncts to antidepressants and for exploring their potential benefits when used adjunctively with psychological therapies to reflect likely clinical usage.

## Data Availability

The original contributions presented in the study are included in the article/Supplementary Material; further enquiries can be directed to the corresponding author.
