# Supplementary material for: Understanding the research landscape of over-the-counter herbal products, dietary supplements, and medications evaluated for depressive symptoms in adults: a scoping review
Source: Front Pharmacol. 2025 Jul 15;16:1609605. doi: 10.3389/fphar.2025.1609605 (PMC12303899; doi:10.3389/fphar.2025.1609605)
Supplement: Supplementary file 1 [file Table1.docx]

**Supplementary file 1: Search terms – Medline example**

| 1 (anxi* or (anxiety adj1 disorder) or (stress* adj1 (psychological or emotional)) or panic attack or depress* or mental health or dysthymia or (panic adj1 disorder) or (sleep adj1 disorder) or insomnia or (mood adj1 disorder) or (psychological adj1 distress)).ti,ab,kw. |
| --- |
| 2 mental health/ or anxiety disorders/ or mood disorders/ or Anxiety/ or exp Depression/ or exp "Sleep Initiation and Maintenance Disorders"/ |
| 3 1 or 2 |
| 4 ("Over-the-counter" or OTC or (over the counter adj1 (medic* or drug or product)) or "General sales" or "pharmacy only" or Non-prescription or Self-medication or Self-prescription or anti-histamines or (botanical or herb* or "medicinal plant" or fungus or medicinal mushroom or herbal tea or plant extract or flower extract or root extract or seed extract or "bach flower" or "natural product" or CBD or cannabidiol) or ("nutritional supplement" or "dietary supplement" or vitamins or minerals or "amino acids" or "essential fatty acids" or "omega-3 fatty acid" or 5-Htp or 5-Hydroxytryptophan or tryptophan or probiotic or prebiotic or melatonin) or ((Traditional adj1 medicine) or (Chinese adj1 medicine) or ayurv* or homeopathy or "homeopathic medicine" or phytotherap* or nutraceutical or "herbal medicine")).ti,ab,kw. |
| 5 exp Nonprescription Drugs/ or plant extracts/ or teas, herbal/ or Plants, Medicinal/ or Cannabidiol/ or Dietary Supplements/ or exp Vitamins/ or Vitamin D/ or Vitamin E/ or Folic Acid/ or magnesium compounds/ or minerals/ or zinc compounds/ or amino acids/ or Tryptophan/ or Fatty Acids/ or homeopathy/ or exp medicine, traditional/ or Melatonin/ |
| 6 4 or 5 |
| 7 exp animals/ not humans.sh. |
| 8 (randomized controlled trial or controlled clinical trial).pt. or randomized.ab. or placebo.ab. or clinical trials as topic.sh. or randomly.ab. or trial.ti. |
| 9 8 not 7 |
| 10 (cost-effectiveness or (cost adj1 effectiveness adj1 analysis) or (cost adj1 benefit adj1 analysis) or "economic evaluations").ti,ab,kw. |
| 11 Cost-Benefit Analysis/ |
| 12 9 or 10 or 11 |
| 13 3 and 6 and 12 |
| 14 limit 13 to humans |
