# Supplementary material for: Understanding the research landscape of over-the-counter herbal products, dietary supplements, and medications evaluated for depressive symptoms in adults: a scoping review
Source: Front Pharmacol. 2025 Jul 15;16:1609605. doi: 10.3389/fphar.2025.1609605 (PMC12303899; doi:10.3389/fphar.2025.1609605)
Supplement: Supplementary file 2 [file Table2.docx]

**Supplementary File 2: Ongoing trials**

| **Investigator  Country Trial type** | **Trial ID** | **Last updated (MMM-YY)** | **Status** | **Depression alone?** | **Product type** | **Product** | **Comparator** | **Target sample size** |
| --- | --- | --- | --- | --- | --- | --- | --- | --- |
| Moschonis RCT Australia | ACTRN12621000675820 | Jun-21 | Not yet recruiting | Yes | Dietary supplement | Probiotics: 355mg of 4x109 CFU of four strains | Placebo | 48 |
| Rush University Medical Centre RCT USA | NCT03317678 | Dec-22 | Complete but publication not found | Yes | Dietary supplement | Probiotics and prebiotics: BioKefir™ lactose-free fermented milk drink containing 12 different species of bacteria plus 2 g of prebiotic fibre (pectin and inulin). | Placebo (non-fermented milk) | 40 |
| Ren RCT China | NCT04567147 | Sep-20 | Complete but publication not found | Yes | Dietary supplement | Probiotic: Five strains each at a dosage of 3.0E+10 CFU per 2g sachet. | Placebo sachet | 89 |
| Ren RCT China | NCT04570072 | Sep-20 | Unknown status | Constipation | Dietary supplement | Probiotic: Five strains each at a dosage of 3.0E+10 CFU per 2g sachet. | Placebo sachet | 200 |
| Lopresti RCT Australia | ACTRN12622001078741 | Jan-24 | Complete but publication not found | Yes | Dietary supplement | Probiotics: Bifidobacterium adolescentis (5 billion colony-forming units) Lacticaseibacillus rhamnosus + Bifidobacterium BB-12 (1 billion colony-forming units) | Placebo | 60 |
| Sharbafchi RCT Iran | IRCT20190404043159N5 | Jan-22 | Recruiting | Irritable bowel syndrome | Dietary supplement | Probiotics and prebiotics: Familact 2 Plus (eight bacterial strains and fructooligosaccharide) + usual treatment | placebo + usual treatment | 48 |
| Lindqvist RCT Sweden | NCT03660280 | Nov-23 | Complete but publication not found | Yes | Dietary supplement | Probiotics | Placebo | 75 |
| Sundram RCT Australila | ACTRN12621000115831 | Feb-21 | Not yet recruiting | Yes | Dietary supplement | Probiotics (Ecologic Barrier – eight strains 5Billion CFU) | Placebo | 20 |
| Yanling RCT China | ChiCTR2100049538 | Oct-22 | Recruitment pending | Ulcerative colitis | Dietary supplement | Probiotics (dose NR) | Placebo | 54 |
| Sampsell & Uzelman RCT Canada | NCT05568498 | Oct-22 | Ongoing | Parkinson's | Dietary supplement | Probiotics (Ecologic® BARRIER 849) | Placebo | 60 |
| Jazayeri  RCT Iran | IRCT201601102394N20 | Feb-18 | Complete but publication not found | Yes | Dietary supplement | Probiotics (two strains) | Placebo | 40 |
| Gawlik-Kotelnicka 4 arm RCT Poland | NCT04756544 https://doi.org/ 10.3390/jcm10071342 | Feb-21 | Unknown status | Half the sample will have Metabolic Syndrome | Dietary supplement | Probiotics (two strains  3x10^9^ CFU | Placebo | 200 |
| Chen RCT Taiwan | NCT04199845 | Dec-19 | Status unknown | Yes | Dietary supplement | Probiotics (PS-128, 300mg twice a day) | Placebo | 60 |
| Bambling  RCT Australia | ACTRN12617000419369 | Nov-18 | Recruiting | Yes | Dietary supplement | Probiotics 20 billion CFU plus magnesium orotate (1600 mg / day) | Placebo | 130 |
| Bansal RCT India | CTRI/2020/05/025027 | Nov-21 | Complete but publication not found | Yes | Dietary supplement | Probiotics (Sporolac plus capsule, containing five strains of 2.5 billion cells) | Placebo | 100 |
| Rucklidge RCT New Zealand | ACTRN12612000647831 | Jan-20 | Withdrawn (reason not reported) | Anxiety | Dietary supplement | Probio'Stick (Lactobacillus Helveticus R0052 and Bifidobacterium longum R0175 (3 x 109 colony-forming units/stick)). 1 stick/day. | Placebo | 63 |
| Milev RCT Canada | NCT03277586 (also protocol publication at https://doi.org/10.1159/000496406) | Oct-24 | Terminated (Covid-19) | Yes | Dietary supplement | Probio'Stick (Lactobacillus Helveticus R0052 and Bifidobacterium longum R0175). | Placebo | 108 (28 achieved) |
| Kious  RCT USA | NCT02922725 | May-22 | Terminated (Covid-19 - descriptive results presented on clinicaltrials.gov in subsample) | Yes | Dietary supplement | 5-hydroxytryptophan and Creatine | Placebo | 54 (32 achieved) |
| Kious 3 arm RCT USA | NCT04395183 | May-24 | Complete but publication not found | Yes | Dietary supplement | 5-HTP + placebo  Creatine + placebo  5-HTP + creatine | Placebo + placebo | 172 |
| Fouladi 3-arm RCT Iran | ACTRN12613001275752 | Jan-20 | Not yet recruiting (unclear accuracy) | Yes | Dietary supplement | 5-HTP 50 mg plus 500 mg L-tyrosin, 500 mg cysteine, 1.5 mg pyrodoxine and 0.05 mg selenium + SSRI  5-HTP 300 mg plus 3000 mg L-tyrosin, 500 mg cysteine, 1.5 mg pyrodoxine and 0.05 mg selenium + SSRI | Placebo + SSRI | 240 |
| Rucklidge RCT New Zealand  NUTRIMUM | ACTRN12617000354381  Doi:[10.1186/s12884-020-03143-z](https://doi.org/10.1186%2Fs12884-020-03143-z) | Apr-24 | Complete but publication not found | Pregnancy (12-24 weeks gestation) | Dietary supplement | Multivitamin: Daily Essential Nutrients (Hardy Nutritionals), | Active control containing iodine and riboflavin | 120 |
| Fakhari  RCT Iran | IRCT138901233695N1 | Feb-18 | Complete but publication not found | Yes | Dietary supplement | Multivitamin: Folic acid 2 mg, 40 mg pyridoxine and 100mcg cobalamin + 20mg fluoxetine | Placebo + fluoxetine | 70 |
| Dyall  RCT UK | NCT04844034 | Jun-23 | Recruiting | Anxiety | Dietary supplement | Multivitamin: 1,125 mg EPA, 441 mg DHA, 330 mg Magnesium, 7.5 mg a-tocopherol | Placebo | 94 |
| Ricca RCT Italy | NCT04832178 | Feb-24 | Recruiting | Yes | Dietary supplement | Multivitamin (SUMOR: SAMe 250 mg, Betaine hydrochloride 250 mg, Vit C 80 mg, Vit B1 1.1 mg, Vit B2 1.4 mg, Vit B6 1.4 mg, Vit B12 2.5 µg, Folic Acid 200 µg, Selenium 37 µg | Placebo | 102 |
| Arunpongpaisal  RCT Thailand | TCTR20161219004 | May-24 | Complete but publication not found | Yes | Dietary supplement | Melatonin 20mg | Placebo | NR |
| Nazari Nasab RCT Iran | IRCT20201125049487N1 | Jan-21 | Complete but publication not found | Yes | Dietary supplement | Omega- 3 fatty acid 300-600mg + SSRI | Placebo + SSRI | 60 |
| Nakano RCT Japan | JPRN-UMIN000013525 | Oct-23 | Recruiting (may not be accurate, reg in 2014 ) | Yes | Dietary supplement | Omega 3 fatty acids | Placebo | 60 |
| Coryell  RCT USA | NCT00256412 | Jun-08 | Complete but publication not found | Yes | Dietary supplement | Omega-3 fatty acids:  1. EPA 0.7 grams/day  2. EPA 1.5 grams/day | Placebo | 24 |
| Jazayeri  RCT Iran | IRCT201411112394N14 | NR | Complete but publication not found | Type 2 diabetes | Dietary supplement | Resveratrol 240mg/day | Placebo | 60 |
| Rao RCT India | CTRI/2020/12/030020 | Nov-21 | Not yet recruiting | Yes | Dietary supplement | Vitamin D3 60000IU/5ml syrup | Placebo | 70 |
| Name not reported RCT Denmark | EUCTR2010-023531-42-DK | Aug-21 | Complete but publication not found | Yes | Dietary supplement | Vitamin D (cholecalciferol 70mcg) | Placebo | 400 |
| Effatpanah  RCT Iran | IRCT2014080315276N2 | Feb-18 | Complete but publication not found | Yes | Dietary supplement | Vitamin D3 (dose NR) + 500mg calcium carbonate + buproprion 450mg | Placebo + 500mg calcium carbonate + buproprion 450mg | 64 |
| Afrin RCT Bangladesh | NCT03754712 | Nov-18 | Unknown status | Vitamin D deficiency | Dietary supplement | Vitamin D3 2000IU per day + SSRI | SSRI alone | 90 |
| Putranto RCT Indonesia | NCT04917458 | Jun-21 | Unknown status | Type 2 diabetes | Dietary supplement | Vitamin D3 4000IU | Placebo | 90 |
| Solati  3 arm RCT Iran | IRCT20110514006480N17 | Nov-18 | Recruiting | Yes | Herbal product | 1. 60 drops of herbal Hypiran drop per day + 20-60mg fluoxetine  2. 60 drops Hypiran + 20-60mg fluoxetine + 8 sessions of mindfulness-based cognitive behavioural therapy | Fluoxetine 20-60mg + M-CBT | 55 |
| Razmi RCT Iran | IRCT20170311033004N3 | Feb-22 | Pending | Yes | Herbal product | Harmaline powder capsules 10 mg | Placebo | 68 |
| Mishra RCT India | CTRI/2018/05/014300 | Nov-21 | Complete but publication not found | Yes | Herbal product | 1. *Withania somnifera* and sesame oil sirodhara 2. Brahmi-Jata yoga + *Bacopa monnieri* and *Nadrostachys jatamansi* 4g for 15 days 4. Combination of both | n/a | 45 |
| Heydari  RCT Iran | IRCT20150309021386N2 | Sep-18 | Recruiting (may not be accurate as 2018) | Type 2 diabetes | Herbal product | 200mg lavender essential oil 4/day + 100mg sertraline | Placebo + 100mg sertraline | 60 |
| Taherzadeh 3 arm RCT Iran | IRCT20130826014477N5 | Sep-18 | Recruiting (may not be accurate, reg in 2018 ) | Post-partum depression | Herbal product | 1. 5mg crocin twice a day  2. 15mg saffron twice a day | Placebo alongside drug regimen | 45 |
| Thukhammee RCT Thailand | TCTR20210407002 | May-24 | Complete but publication not found | Depression and/or anxiety | Herbal product | *Anethum graveolens* Linn. (Dill) beverage 1000mg/day | Placebo beverage | 60 |
| Kuan-Pin RCT Taiwan | NCT04002219 | Oct-22 | Complete but publication not found | Yes | Herbal product | *Cordyceps militaris* as beverage (dose NR) | Placebo | 80 |
| Steels RCT Australia | ACTRN12622000777796 | Mar-24 | Stopped early (publication not found) | Yes | Herbal product | *Curcuma longa* rhizome (6.4g), *Boswellia serrata* resin (1.5 g), *Bupleurum falcatum* root (750 mg), and *Centella asiatica* leaf (1.5 g). 4 tablets per day. | Placebo | 120 |
| Schlatman Pilot three arm RCT Canada | NCT05041647 | Nov-23 | Recruiting | Insomnia | Herbal product | 1. High CBD 1ml (50 mg/ml CBD and 2 mg/ ml THC),  2. Low CBD 1ml (10 mg/ml CBD and 2 mg/ ml THC) | Placebo | 60 |
| Lim RCT South Korea | KCT0003649 | Mar-19 | Complete but publication not found | Stress (depression or anxiety) | Herbal product | *Hydrangea pulcis* molium 500mg/day | Placebo | 100 |
| Contact not reported RCT USA | NCT00042380 | Mar-08 | Complete but publication not found | Yes | Herbal product | Novasoy (soya isflavones) 270mg/day | Placebo | 120 |
| Kim  RCT Korea | KCT0007161 | Sep-22 | Recruiting | Anxiety | Herbal product | SB-109 (*Platycodon grandiflorum* extract and *Poncirus trifoliate* extract). 2 x 600mg/day | Placebo | 150 |
| Talebi RCT Iran | IRCT20171228038114N1 | Sep-18 | Recruiting | Yes | Herbal product | *Cuscuta epithymum* (dose NR) + 50mg sertraline | Placebo + 50mg sertraline | 60 |
